# Innovation in mRNA Vaccines and RNAi via Protein Nanocages

**DOI:** 10.3390/vaccines13060653

**Published:** 2025-06-18

**Authors:** Sohrab Ahmadivand

**Affiliations:** Faculty of Veterinary Medicine, Ludwig-Maximilians-Universität München, 80539 Munich, Germany; sohrab.ahmadivand@lmu.de

**Keywords:** protein nanocages, siRNA delivery, mRNA vaccines, vaccine platform, ferritin, virus

## Abstract

Self-assembling protein nanocages (SAPNs) are distinct natural structures formed by the self-assembly of identical subunits, providing a highly efficient platform and a novel strategy for vaccine development and RNAi therapy. Their internal cavity allows for precise cargo encapsulation, while the externally modifiable surface supports multivalent antigen presentation, thereby enhancing stability, targeted delivery, and immune activation. In addition to serving as stable subunit vaccines with multivalent antigen display, SAPNs can be incorporated into mRNA vaccines (SAPN-RNA vaccines) by pre-fusing with the antigen. This strategy stabilizes secreted antigenic proteins with prolonged presentation to the immune system, and improves vaccine efficacy while reducing off-target effects and minimizing required doses. Additionally, SAPNs can overcome cellular uptake barriers, enhance DNA vaccine efficacy, and enable the co-delivery of antigens and adjuvants. Functionalization with adjuvants or targeting ligands further improves their immunostimulatory properties and specificity. The SAPN-RNAi strategy optimizes siRNA delivery by promoting lysosomal escape, enhancing targeted uptake, and protecting siRNA from degradation through SAPN encapsulation. This review examines the structural and functional properties of protein nanocages and their applications in vaccine design and RNAi delivery, emphasizing their synergistic effects, and exploring current progress, challenges, and future directions. In conclusion, SAPNs represent a versatile multifunctional platform with broad applicability across subunit, mRNA and DNA vaccines, adjuvant co-delivery, and RNAi therapeutics, with significant potential against viral infections.

## 1. Introduction

RNA biopharmaceuticals are an emerging class of therapies showing significant promise for the treatment and prevention of a wide range of diseases. mRNA vaccines, in particular, offer great potential for both human and animal diseases [1,2]. However, to maximize their efficacy, it is crucial to modulate immunogenicity, developing safe and efficient delivery systems, and stabilizing RNA against degradation. Additionally, cold-chain requirements and biocompatibility concerns hinder large-scale clinical deployment [3,4].

Similarly, RNA interference (RNAi) offers a targeted and highly specific therapeutic strategy to manage viral infections and other diseases by silencing target gene expression. This flexibility provides a distinct advantage over traditional antiviral treatments. Nevertheless, RNAi therapies face significant barriers in clinical translation, primarily related to toxicity, off-target effects, and inefficient delivery to target cells [5,6].

The multivalent display of structurally defined antigenic epitopes on the surface of self-assembling nanostructures such as virus-like particles (VLPs) and self-assembling protein nanocages (SAPNs), constitutes a highly effective strategy for enhancing antigen stability, immunogenicity, and the induction of robust, targeted immune responses. VLPs, derived from viral structural proteins, mimic the size, shape, and surface organization of native virions but are rendered non-infectious due to the absence of viral genomic material. In contrast, SAPNs are naturally occurring, highly ordered, biocompatible protein nanostructures formed through the spontaneous self-assembly of identical subunits. The protein nanocages exhibit exceptional physicochemical properties, including high thermal and pH stability, intrinsic biodegradability, and reversible assembly and disassembly under physiological conditions [7,8]. These characteristics underpin the broad applicability of SAPNs across various biomedical fields, including targeted drug delivery, vaccine development, molecular diagnostics, and therapeutic interventions, particularly when integrated with advanced molecular imaging techniques [9].

Their unique architecture features an internal cavity capable of precise cargo encapsulation, coupled with a modifiable surface that enables multivalent display of ligands or antigenic epitopes [10,11]. This dual functionality facilitates targeted delivery, controlled release, and potent immune activation, positioning SAPNs as versatile platforms for vaccine development and RNAi therapeutics [11,12,13]. Unlike VLPs, which mimic viral structures but require lipid membranes leading to structural variability, SAPNs serve as robust scaffolds that can replace viral envelopes, overcoming challenges associated with VLP assembly and characterization [14,15].

SAPNs were initially explored for subunit vaccines to improve antigen stability and immunogenicity, while supporting DIVA (Differentiating Infected from Vaccinated Animals) strategies and rapid vaccine design [16,17]. Fusing antigens to protein nanocages enhances their solubility in prokaryotic expression systems, likely by promoting proper folding, preventing aggregation, and shielding hydrophobic regions [15]. This improved structural presentation not only facilitates expression but also contributes to enhanced vaccine safety, as the biocompatible nature of SAPNs helps mitigate the antigen-associated toxicity often observed with unstable or poorly expressed proteins [15,18].

In mRNA vaccines, SAPNs fusion (referred to here as SAPN-RNA vaccines) allow for multivalent antigen presentation, which stabilizes antigenic proteins, improves intracellular trafficking, and potentially prolongs antigen expression. These effects lead to more potent and sustained immune responses, including high-affinity neutralizing antibodies at lower doses [19,20]. Similarly, these protein nanocages address challenges related to poor antigen expression and inefficient cellular uptake in DNA vaccines, thereby enhancing their overall immunogenicity [21].

Clinical trials of SAPN-based subunit vaccines targeting influenza, HIV, EBV, and SARS-CoV-2 have demonstrated strong immunogenicity and favorable safety profiles [16,22]. SAPN-RNA vaccines, such as the mRNA (NCT05001373 and NCT05414786) and saRNA HIV candidates utilizing lumazine synthase nanocages, further underscore the promise of this approach in next-generation vaccine development [23,24].

Beyond structural antigen display, SAPNs enable co-delivery of antigens and adjuvants [25,26,27]. This feature enhances immune responses while minimizing off-target effects and systemic toxicity. The multivalent surface display of adjuvant proteins, such as Flagellin, a TLR5 agonist, potentiates immune activation, while encapsulating small/molecule adjuvants within the internal cavity ensures sustained release, thereby improving vaccine efficacy [10,25,26,27].

SAPNs also enhance RNAi delivery by overcoming biological barriers such as instability, nuclease degradation, and endosomal entrapment [28]. Their reversible disassembly (e.g., ferritin) and internal targeting domains (e.g., encapsulin TPs) allow for efficient cargo loading [8,10,11]. Recent studies have shown that SAPNs can protect siRNAs from degradation, promote lysosomal escape, and enhance targeted delivery, resulting in improved gene silencing efficiency [13,29,30,31].

These multifunctional capabilities position SAPNs as a novel and promising platform for RNAi therapy and vaccine development, particularly for mRNA vaccines. This review comprehensively analyzes the structural features, functional mechanisms, and biomedical applications of SAPNs in mRNA, DNA, and subunit vaccines, RNAi delivery, and adjuvant functionalization. It also addresses key design principles, technical challenges, and innovative strategies to facilitate the clinical translation of SAPNs, with a focus on viral infections.

## 2. Self-Assembling Protein Nanocages (SAPNs)

### 2.1. Characteristics

Self-assembling protein nanocages (SAPNs), naturally occurring in various organisms, have emerged as promising platforms for vaccine development and therapeutic protein delivery, including RNAi. These nanoscale structures (10–100 nm) closely mimic the size and shape of viruses, enabling the multivalent display of antigens in highly ordered architectures with up to 240 identical subunits [12,20]. They exhibit exceptional thermal and pH stability, monodispersity, uniform size, biodegradability, biocompatibility, and reversible spontaneous assembly/disassembly [16,22]. Therapeutic molecules can be encapsulated within SAPNs, while engineered surfaces enable antigen display and targeted delivery. Modifying intersubunit interactions allows for modulation of self-assembly and controlled molecular release [32].

The current studied protein nanocages include ferritin, lumazine synthase (LuS), Encapsulin, E2 protein (E2p), and small heat-shock proteins (sHSP), each possessing distinct structural and physiochemical characteristics. They are generally non-immunogenic, and those derived from bacterial sources are preferred to mitigate the risk of autoimmunity [15,33].

Ferritin is a conserved intracellular iron-storage protein and a well-characterized SAPN used in vaccine and drug delivery [17,34]. In mammals, it consists of heavy (21 kD) and light (19 kD) chains, while teleost fish have an additional medium chain (20 kD). Bacterial and plant ferritins contain a single subunit resembling the vertebrate heavy chain [35,36]. Despite up to 80% sequence variation, ferritin maintains a conserved structure, assembling into an octahedral (432) symmetry nanocage of 24 subunits with internal and external diameters of 8 nm and 12 nm [34]. Toxicological studies show ferritin nanoparticles are biocompatible, with no cytotoxicity observed in zebrafish liver (ZFL) cells up to 100 µg/mL [15] and no adverse effects in vivo at imaging doses [35]. Ferritin also exhibits exceptional thermal and pH stability, tolerating temperatures up to 100 °C and pH ranges from 3 to 10, with reversible disassembly below pH 3.4 [8,36]. This structure provides dual functionality: encapsulating internal cargo and displaying multivalent antigens on the external surface via the N-terminus, with *Helicobacter pylori* being the most studied origin of some in clinical trial [17].

*Lumazine synthase* (LuS) is a self-assembling protein nanocage involved in riboflavin biosynthesis, forming capsids composed of 60 subunits arranged into 12 pentameric units [37,38]. With both N- and C-termini exposed on the outer surface and a greater number of subunits compared to ferritin, LuS facilitates efficient antigen attachment, potentially enhancing antigen presentation and immunogenicity. Among the LuS variants, those from *Aquifex aeolicus* (AaLuS) and *Bacillus subtilis* (BsLuS) have been most studied. AaLuS, one of the most thermostable proteins known, has a melting temperature of 120 °C, making it ideal for high-temperature applications [37]. While BsLuS forms smaller capsids, AaLuS can assemble larger shells composed of 180 or more subunits, offering greater versatility in encapsulation [38]. Despite these promising features, LuS remains underexplored, with research primarily focused on pathogens like HIV, SBV, SARS-CoV-2, and RVFV [12,20].

Encapsulins are another self-assembling, icosahedral protein nanocage found in bacteria and archaea, typically composed of 60 subunits, with some variants, like those in *Quasibacillus thermotolerans*, forming larger cages of up to 240 subunits. These protein shells are highly stable under varying pH and temperature conditions, ranging in size from 20 to 42 nm, and can incorporate diverse cargos, including proteins, therapeutic RNA, and adjuvants [39,40]. Encapsulins are classified into four families based on cargo type and operon structure, with computational analysis, of which Family 1 is the most extensively characterized [41]. Encapsulin shell protomers share structural similarity with the HK97 bacteriophage capsomere gp5 and comprise three conserved domains: the axial (A-domain), peripheral (P-domain), and extended loop (E-loop), with the E-loop regulating subunit arrangement and triangulation. Cargo proteins are attached via targeting peptides (TPs) at the C-terminus or, in some cases, the N-terminus, as seen in Firmicute bacteria [42]. Encapsulins can undergo reversible disassembly and reassembly, with the smallest T = 1 (60-mer) T. maritima encapsulin nanocage demonstrating the most stable and effective reassembly after disassembly. It can withstand up to 3 M GuHCl, pH 12, and 90 °C, with reversible unfolding induced by basic pH or GuHCl, making it suitable for encapsulation applications. However, repeated cycles of disassembly and reassembly may result in significant loss of structural integrity [8]. Their simplicity enables efficient production in *E. coli*, and by fusing antigen-encoding genes to the C-terminal region, they have shown potential as a vaccine platform against infectious pathogens such as influenza [43], SARS-CoV-2 [44], and rotavirus [45].

Dihydrolipoyl Acetyltransferase (E2 protein; E2p) from *Geobacillus stearothermophilus* is another protein nanocage that forms a 27 nm icosahedral structure with 60 subunits and 532-fold symmetry [46]. However, some variant forms of octahedral E2p consist of 24 identical polypeptide chains arranged with 432 symmetries [46].

As a key component of the pyruvate dehydrogenase complex, E2p transfers the acetyl group to CoA. Gram-negative bacteria like *E. coli* form 24-subunit structures, while yeast and mammals form the 60-subunit pentagonal dodecahedron, with catalytic domains creating cavities for binding lipoyl groups and CoA [47]. E2p nanocages offer high stability, ease of expression, and molecular encapsulation. Dalmau et al. [48] engineered E2p for expression in E. coli, demonstrating exceptional thermostability (unfolding onset: 81.1  ±  0.9 °C, midpoint: 91.4  ±  1.4 °C) while preserving its dodecahedral structure. Peng et al. [49] also developed a pH-responsive E2p nanocage incorporating a switchable GALA peptide, which disassembles at pH 7.0 and reassembles at pH 4.0 in a reversible manner. These modifications enable non-native functionality and cargo encapsulation. The N-terminus of truncated E2p, retaining only the catalytic domain, remains accessible for antigen fusion, facilitating the presentation of target antigens. E2p has been explored as a platform for biomedical applications, successfully displaying multivalent antigens from infectious viruses such HIV-1 gp140, Ebola glycoprotein, modified SARS-CoV-2 RBD, and has even shown promise in malaria transmission-blocking vaccines [50]. Its unique structure and larger size enhance E2p versatility as both an antigen presentation and therapeutic cargo delivery platform.

Small heat-shock proteins (sHSPs) are found in a wide variety of organisms and are unique for being ATP-independent. sHSPs consist of three domains: the N-terminal domain (NTD), the α-crystallin domain (ACD), and the C-terminal domain (CTD), which regulate the assembly of monomers (13–43 kDa) into large oligomers (up to 40 subunits) [51]. They form nanoparticles ranging from 12 to 20 nm in diameter, typically composed of 12 or 24 subunits [52]. They can be easily modified for antigen display by exposing either the N-terminus (e.g., M. tuberculosis HSP16.3) or the C-terminus (e.g., *M. jannaschii* HSP16.5) [53]. These nanocages also exhibit intrinsic protective effects against respiratory viruses, making them promising candidates for vaccine platforms [54]. The sHsp nanocages have eight pores (3 nm and 1.7 nm) for molecular trafficking and exhibit high stability [55]. The archaeal sHsp from *M. jannaschii* forms a 12 nm nanocage, tolerating up to 70 °C and pH 5–11 [56]. sHSP nanocages have diverse biomedical applications. However, their intrinsic immunogenicity requires careful evaluation to avoid autoimmunity, particularly with homologous applications [57].

Given these characteristics, SAPNs represent an efficient strategy to enhance the immunity and stability of subunit and nucleic acid-based vaccines, as well as for therapeutic delivery (Table 1 and Table 2). Further studies are essential to explore and characterize additional protein nanocages with optimized designs, to assess long-term efficacy and facilitate regulatory approval, ensuring their widespread adoption in disease control strategies.

### 2.2. Production

Protein nanocages provide a versatile platform for antigen presentation, with most architectures orienting the C- and N-termini toward the interior and exterior, respectively. Antigen display on the nanocage surface has been successfully implemented through genetic fusion, chemical conjugation, and protein ligation systems [17]. While genetic fusion to these termini enables precise antigen incorporation, optimizing structural design is crucial to maintaining nanoparticle stability and assembly integrity, particularly for larger or more complex antigens. Chemical conjugation, though effective, is a complex procedure that requires specific conditions and reagents, potentially resulting in heterogeneous decoration that hinders efficient immune presentation [59].

The SpyTag-SpyCatcher system, a novel technology for vaccine development, offers a solution by enabling highly efficient coupling of antigens to the platform while preserving their native conformation. This system utilizes the Streptococcus pyogenes fibronectin-binding protein FbaB, split into SpyCatcher (113 aa) and SpyTag (13 aa), to form an irreversible peptide bond between the NP and antigen, regardless of antigen size [60].

The expression and purification of the protein nanocages are critical steps to ensure high-quality nanoparticles with consistent structural and immunological properties. SAPNs can be produced using bacterial, mammalian, or insect cells, depending on the complexity of the protein and required post-translational modifications. Mammalian systems, such as HEK293F and ExpiCHO, ensure proper folding and glycosylation, which are crucial for antigenicity in ferritin-based nanoparticles displaying viral antigens like HIV gp120 or SARS-CoV-2 spike proteins [12]. Baculovirus expression systems provide high yields with appropriate modifications, enabling the successful development of SAPN-based vaccines, including ferritin, Lus, and E2p [61,62]. Bacterial expression systems offer a cost-effective, efficient platform for SAPN production, with high yields and rapid synthesis. Genetic fusion with nanocage platforms like ferritin enhances solubility and stability, while optimization strategies such as molecular chaperones and RNA-Interaction Domains (RIDs) improve protein folding and assembly efficiency, making bacteria an ideal choice for scalable SAPN vaccine development [63].

Purification typically involves affinity chromatography, size-exclusion chromatography, or ultracentrifugation to isolate fully assembled nanoparticles. For instance, ferritin NPs displaying SARS-CoV-2 spike proteins are purified via nickel-affinity chromatography followed by size-exclusion to remove unassembled or aggregated particles, ensuring a homogenous population with optimal antigen presentation capabilities [12]. In bacterial systems, recovery from inclusion bodies may require denaturation and refolding, followed by dialysis to remove salts and stabilize nanoparticles for downstream applications. Optimizing factors like protein concentration, pH, ionic strength, and temperature during expression and purification is essential to achieve high-yield assembly of SAPNs, as demonstrated in ferritin nanoparticles, which require divalent cations like Fe^2^⁺ for proper self-assembly [14].

Recent advances focus on optimizing the design and production of SAPN-based vaccines to enhance their scalability and efficacy. Tyrosinase-mediated bioconjugation enables site-specific attachment of antigens such as the SARS-CoV-2 spike receptor binding domain to ferritin nanoparticles while preserving antigen integrity and allowing controlled multivalent display [64]. Concurrently, the strategic insertion of polyhistidine tags into structurally permissive loops of *H. pylori* ferritin facilitates efficient immobilized metal affinity chromatography (IMAC)-based purification, with purification yield dependent on tag location and antigen size—the His-4 site proving optimal for spike protein constructs [65]. These innovations represent critical steps toward the streamlined development and manufacturing of SAPN-based vaccines.

Beyond subunit vaccines (SAPN-Subunit), SAPNs can also be incorporated into nucleic acid vaccines, such as RNA and DNA vaccines, by genetically fusing the antigen, resulting in the secretion of stable antigenic proteins from host cells in a multivalent display manner that enhances immune responses. SAPNs further enable the co-delivery of protein antigens, adjuvants, RNA, and other cargo, either within the internal cavity or on the external surface.

Early strategies for internal cavity loading involved engineering the nanocage’s C-terminus for cargo encapsulation. Encapsulins exemplify drug delivery by enabling in vivo co-packaging of proteins through selective mechanisms such as targeting domains (TDs), transporter peptides (TPs), or RNA-binding domains [11]. Other SAPN loading methods include biomineralization, passive diffusion, and disassembly/reassembly [66]. Biomineralization is effective for synthesizing metal oxide nanoparticles but has limited applicability for other drugs. Passive diffusion allows small molecules to enter ferritin without compromising structural integrity, whereas disassembly/reassembly enables loading of larger compounds by dissociating nanocages at pH 2 and reassembling at pH 7. Alternative methods, such as urea-induced (8M urea) or hydrostatic pressure (450 MPa) disassembly, provide controlled loading and release [66].

However, reassembly rates vary among nanocages, with ferritin showing the highest efficiency, while others, such as encapsulin (T = 3), may exhibit lower reassembly or lack the ability to reassemble completely [8]. Moreover, the recovery of reassembled proteins can be suboptimal [7].

**Figure 1:**
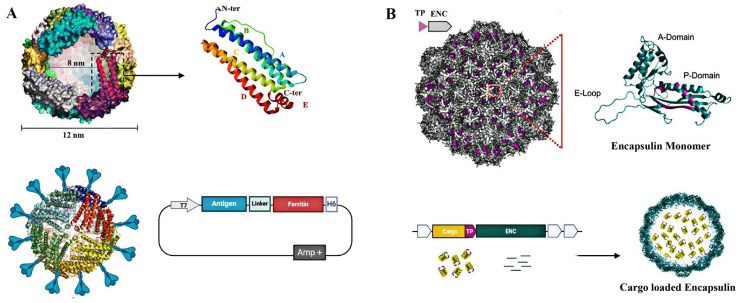
Self-assembling protein nanocages (SAPNs) for multivalent antigen display and cargo encapsulation. (**A**) Schematic representation of ferritin nanocages and a monomeric subunit. Ferritin assembles into a 24-subunit spherical cage with inner and outer diameters of approximately 8 nm and 12 nm, respectively. Multivalent antigen display can be achieved via genetic fusion of antigen-encoding sequences to the C-terminal region of ferritin subunits. (**B**) Schematic illustration of a core encapsulin operon and targeting peptide (TP)-mediated cargo encapsulation. Encapsulin monomers self-assemble into icosahedral protein cages ranging from 20 to 42 nm in diameter, composed of 60, 180, or 240 identical subunits. A domain-level view of the encapsulin monomer is shown. Antigen display on the encapsulin surface can also be achieved through C-terminal genetic fusion. Created with BioRender.com.

The external surface of SAPNs can be modified for targeted delivery, allowing precise control over the location and timing of payload release or immune activation. Surface modifications such as the KALA peptide and L17E peptide have been employed to functionalize SAPNs for tumor therapy, thereby enhancing targeted delivery and cellular uptake [13,67]. To significantly enhance vaccine efficacy, one effective strategy is the incorporation of dendritic cell-targeting ligands such as CD40 and lymphotactin (XCL1). These ligands can be conjugated to SAPNs using the SpyTag-SpyCatcher system or chemical methods to facilitate targeted delivery. Together, these strategies highlight the versatility of SAPNs as multifunctional platforms for vaccine and therapeutic delivery (Figure 1).

## 3. Self-Assembling Protein Nanocages (SAPNs) as Vaccine Platform

### 3.1. SAPN-Subunit Vaccines

Subunit vaccines offer a safer alternative to traditional whole-virus vaccines by utilizing purified viral antigens, reducing the risks associated with live virus replication. Despite their safety, subunit vaccines can have variable efficacy, necessitating strong adjuvants and repeated administration. Additionally, due to GI instability, injection is required, making mass administration costly, labor-intensive, and stressful, particularly in veterinary medicine, where the vaccination of large animal populations presents significant challenges [68,69].

**Figure 2:**
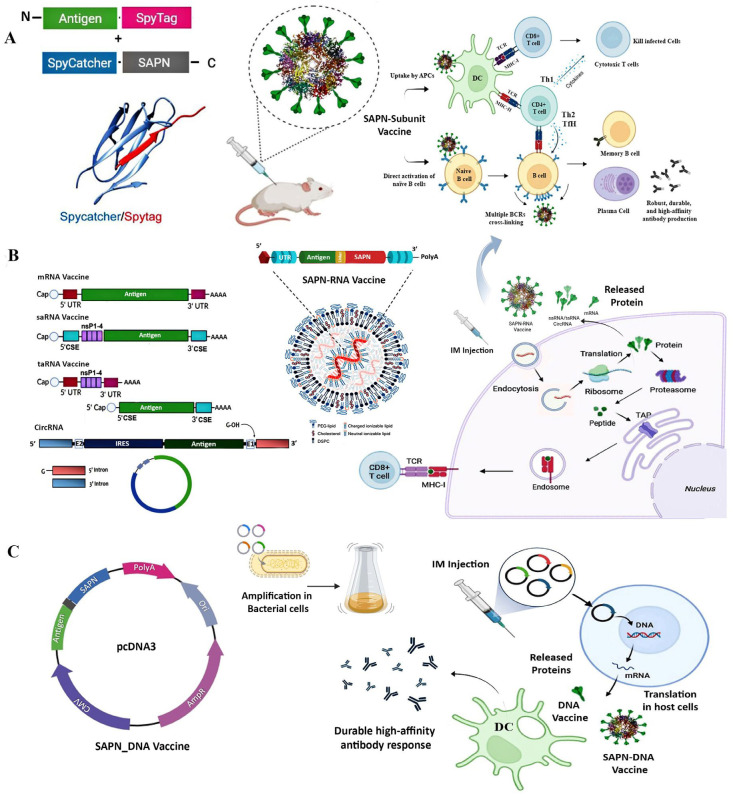
Self-assembling protein nanocages (SAPNs) as versatile platforms for subunit, RNA, and DNA vaccines. (**A**) SAPN-Subunit Vaccines: antigens are covalently linked to protein nanocages using the SpyTag/SpyCatcher system, enabling a highly ordered, multivalent display with enhanced stability. This promotes robust immune responses by directly activating naïve B cells through efficient BCR cross-linking and facilitating antigen uptake by antigen-presenting cells (APCs) such as such as dendritic cells (DCs), leading to strong cellular immunity and durable, high-affinity antibody production. (**B**) SAPN-RNA Vaccines: RNA platforms, including conventional mRNA, self-amplifying RNA (saRNA), trans-amplifying RNA (taRNA), and circular RNA (circRNA), encode SAPN-antigen fusion proteins. Delivered via lipid nanoparticles (LNPs), these RNAs are translated in situ, generating stabilized antigens that enhance immune presentation, cellular uptake, and overall vaccine efficacy while reducing off-target effects and required doses. (**C**) SAPN-DNA Vaccines: plasmid DNA encoding SAPN–antigen fusions is delivered intramuscularly (IM), leading to in situ antigen production within host cells. This forms multivalent antigen displays with enhanced stability, cellular uptake, and immunogenicity. The SAPN platform is based on lumazine synthase, which self-assembles into 60-subunit icosahedral nanocages (~16 nm in diameter; PDB ID: 8F25). Created with BioRender.com.

Through the SAPN-Subunit vaccine approach, self-assembling protein nanocages can be engineered for subunit vaccines by modifying the external surface for multivalent antigen display via genetic fusion, chemical conjugation, or protein ligation, with the potential for targeted delivery [16,17,70]. These vaccines are DIVA-compatible and can be produced in a variety of expression systems, including both prokaryotic and eukaryotic systems, for large-scale production. They outperform subunit vaccines by efficiently displaying complex antigenic proteins from enveloped viruses and rescuing insoluble antigens [15]. The approach also enhances the immunogenicity and stability of subunit vaccines through their repetitive nanostructure, facilitating both oral delivery and mass administration. The unique nanoscale size (<50 nm) and multivalent structure of SAPN-subunit vaccines mimic viral particles, promoting enhanced immune activation (Figure 2A). SAPNs improve antigen uptake by antigen-presenting cells (APCs) and the direct activation of naïve B cells, and enable efficient cross-linking of B-cell receptors (BCRs), inducing innate immune responses via pattern recognition receptors (PRRs) [71,72].

They engage MHC-I and MHC-II pathways, triggering both cellular and humoral responses with Tfh cell activation, which drive germinal center responses essential for generating durable, high-affinity antibody responses [19].

SAPN-Subunit vaccines have emerged as a promising approach with significant potential for human and veterinary applications. Ferritin nanocages, first used in H1N1 influenza vaccines in 2013, have since been developed for influenza, HIV, EBV, and SARS-CoV-2, with several candidates currently in human clinical trials, particularly following the SARS-CoV-2 pandemic in 2021 [17]. Notably, three ferritin-based influenza vaccines (NCT03186781, NCT04579250 and NCT03814720) have reached phase I trials, demonstrating favorable safety profiles and the ability to induce broadly neutralizing antibody responses [73,74,75].

For veterinary use, ferritin vaccines have been explored for economically significant swine respiratory viruses, including swine influenza virus, classical swine fever virus (CSFV) [76], porcine deltacoronavirus (PDCoV) [62], and porcine reproductive and respiratory syndrome virus (PRRSV) [77]. These vaccines offer strain cross-protection and induce stronger humoral and cellular immune responses compared to commercially available inactivated or subunit vaccines [17]. A ferritin-based nanovaccine displaying the H1HA stem region induced broad cross-reactive antibodies, providing full protection against lethal H5N1 in mice and partial protection in ferrets [78]. In comparison to conventional swine influenza vaccines, ferritin-based vaccines triggered stronger immune responses, including higher hemagglutinin inhibition titers and robust IgG production. This vaccine offered complete protection against H1N1 and reduced lung damage in mice and piglets, even against a heterologous strain (81% homology), with cross-protection against H1N1 and H3N2 in mice [79]. A self-assembled nanoparticle African swine fever (ASF) vaccine was designed to target dendritic cells by coupling a 24-mer ferritin scaffold with dominant ASFV epitopes (p72, CD2v, pB602L, p30) and fusing it to the chemokine XCL1 receptor via the SpyTag/SpyCatcher system. Compared to monomeric protein forms, the vaccine elicited significantly stronger T and B cell responses in mice, with ASFV-specific antibody responses sustained for over 231 days [80].

Recently, in the first study for non-mammalian viruses, the ferritin platform was evaluated against infectious hematopoietic necrosis virus (IHNV), an enveloped WOAH-notifiable rhabdovirus affecting aquaculture, demonstrating potential for controlling fish viruses and its high stability for oral mass delivery [15].

Lumazine synthase (LuS) and E2 protein nanocages have been investigated as platforms for presenting complex viral antigens in vaccines, enhancing neutralizing responses. For instance, an AaLuS-based Schmallenberg virus (SBV) vaccine provided 80% protection in mice and induced sterile immunity in cattle with a single dose [81]. When conjugated with the SARS-CoV-2 spike protein, the LuS vaccine elicited stronger neutralizing responses compared to the spike protein alone [82]. Additionally, the LuS vaccine activated VRC01-class B cells, fostering a broadly neutralizing response against HIV-1 [83]. The IAVI G001 trial (NCT03547245) is an important early-stage study in the HIV germline-targeting vaccine strategy, utilizing LuS nanocages. SARS-CoV-2 vaccines using ferritin and E2p nanocages displaying HR2-deleted glycine-capped spike (S2GΔHR2) proteins generated high neutralizing antibody titers and robust T cell responses, including Th1 and memory CD4+ cells. E2p-based vaccines, in particular, demonstrated improved immunogenicity at lower doses [84]. Furthermore, a LuS-subunit vaccine using the Gn head domain provided protection against viremia and clinical signs in mice and lambs infected with Rift Valley fever virus (RVFV), with complete protection achieved in lambs when conjugated to E2p nanocages [85].

Encapsulin, another SAPN, has shown promise for vaccines against rotavirus, influenza, and SARS-CoV-2. Encapsulin from Myxococcus xanthus, engineered with the SpyTag-SpyCatcher system to display 180 copies of the SARS-CoV-2 spike RBD, demonstrates strong antigenicity and thermotolerance. When adjuvanted with SWE, it elicits potent neutralizing antibody titers against multiple variants, including Omicron [44]. Encapsulin-based influenza vaccines (180 copies) outperformed ferritin formulations (24 copies), emphasizing the impact of nanocage architecture on immunogenicity [43]. For rotavirus, a 60-mer encapsulin vaccine induced strong VP8*-specific IgG responses and neutralizing antibodies, surpassing subunit vaccines in mice and balancing Th1/Th2 immune responses [45].

The study by Choi et al. [86] demonstrated that *T. maritima* encapsulin nanocages fused with the OT-1 peptide enabled effective antigen delivery to dendritic cells, resulting in the activation of antigen-specific cytotoxic CD8⁺ T cells. In a melanoma mouse model, vaccination with OT-1-Encaps led to significant tumor suppression.

Small heat shock proteins (sHSPs) have also shown potential for durable antibody production. In mice, intranasal OVA-sHSP vaccines accelerated IgG1 and mucosal IgA responses, improving lung immunity and influenza challenge outcomes [54].

Overall, SAPN-subunit vaccines have demonstrated significant potential in both human and veterinary medicine, offering enhanced antigen presentation, strong immune responses, cross-protection, and stability for oral delivery. Beyond subunit vaccines, SAPNs can also be integrated into nucleic acid vaccine platforms (mRNA and DNA) and utilized for RNAi delivery, with multiple advantages (Figure 2 and Table 3).

### 3.2. SAPN-RNA Vaccines: Synergizing mRNA Vaccines with Protein Nanocages

#### 3.2.1. mRNA Vaccines and Mechanisms

Messenger RNA (mRNA) vaccines are an innovative platform for vaccine development, offering rapid production, scalability, and the ability to target a wide range of pathogens and diseases as demonstrated during the SARS-CoV-2 pandemic. LNP delivery systems effectively protect mRNA, facilitate cellular uptake, and enhance immune responses through Tfh cell activation, supporting high-affinity antibody production [20,92]. They are categorized into four main types: conventional mRNA vaccines, self-amplifying RNA (saRNA) vaccines, trans-amplifying RNA (taRNA) vaccines, and circular RNA (circRNA) vaccines (Figure 2B).

Conventional mRNA vaccines are based on synthetic mRNA transcribed in vitro, incorporating stabilizing features such as 5′ capping, untranslated regions (UTRs), and poly(A) tails to optimize translation and prevent degradation. Advances such as anti-reverse cap analogs (ARCAs) and modified nucleotides like N1-methylpseudouridine have improved mRNA stability and reduced unintended immune activation, as demonstrated in COVID-19 vaccines [93].

In contrast, saRNA vaccines incorporate replication machinery derived from alphaviruses, including nonstructural proteins (nsP1–4), enabling intracellular RNA amplification [94]. This self-amplifying feature reduces the required mRNA doses and lowers production costs while prolonging the expression of the encoded antigen, lasting up to 86 days [95], while mRNA vaccines show reduced expression within two weeks [4]. saRNA vaccines also elicit type I interferon (IFN-I) responses, enhancing adaptive immunity without integrating into the host genome, ensuring a safer profile [96]. However, the longer RNA sequence of saRNA presents challenges in stability and manufacturing. The taRNA vaccines address this by separating the replicase and antigen-encoding sequences onto distinct mRNA molecules, improving translation efficiency while reducing the challenges posed by long RNA constructs [97].

Meanwhile, circRNA vaccines, with their covalently closed structure, offer enhanced stability and prolonged antigen expression, as they are resistant to exonucleases. Despite lacking a 5′ cap and poly(A) tail, translation in circRNA vaccines is facilitated by internal ribosome entry sites (IRESs), leading to sustained protein production with reduced immune activation, positioning circRNA as a promising platform for future vaccine development [98].

Recent studies have revealed the potential of mRNA vaccines as a promising strategy for immunization against diverse human and zoonotic pathogens beyond SARS-CoV-2, such as influenza, dengue virus, HSV-2, rabies, and Zika virus, with distinct advantages compared to other platforms [2].

mRNA vaccines elicit strong humoral and cellular immunity by directing antigen translation in the cytosol. Antigen presentation via MHC-I activates cytotoxic CD8+ T cells, while MHC-II processing stimulates CD4+ T cells and enhances B cell antibody production, ensuring a robust and durable immune response [4].

mRNA vaccines are also gaining attention in veterinary medicine as innovative solutions for managing viral infections in farm animals [1]. They elicit strong humoral and cellular immunity, often surpassing conventional vaccines in swine, poultry, and companion animals. Studies on foot-and-mouth disease virus (FMDV), bovine viral diarrhea virus (BVDV), ASFV, porcine reproductive and respiratory syndrome virus (PRRSV), and avian influenza virus (AIV), demonstrate high neutralizing antibody titers and durable protection [99].

mRNA vaccines are also emerging as a promising approach to control viral diseases in aquaculture, a major and ongoing industry challenge [100,101,102], showing encouraging results despite the unique and simpler immune systems of aquatic species [103,104].

By targeting zoonotic pathogens, mRNA vaccines provide a proactive approach to reducing the risk of animal-to-human transmission, supporting One Health strategies [1].

While mRNA vaccines have shown success against viruses and cancers, they face challenges in producing bacterial proteins, particularly due to issues with folding, transport, and post-translational modifications. Their inability to produce complex antigens, such as polysaccharides, limits their competitiveness with traditional bacterial vaccines [105].

Overall, mRNA vaccines show great promise for human and animal diseases, but optimizing immunogenicity, delivery, and stability is essential to improve efficacy and address challenges like cold-chain storage and biocompatibility.

#### 3.2.2. Self-Assembling Protein Nanocages for mRNA Vaccines Development

mRNA vaccines elicit strong humoral and cellular immunity by mimicking a viral infection. Once inside host cells, the mRNA is translated into protein, which can either function intracellularly and/or be secreted. Cytoplasmic proteins are degraded by proteasomes into peptides that are presented on MHC class I molecules, activating CD8+ T cells. Secreted proteins are taken up by APCs, processed, and presented via MHC class II [4] (Figure 2B). Moreover, cross-presentation allows exogenous antigens to be processed and displayed on MHC class I, further enhancing CD8+ T cell responses [106]. The mRNA-LNP formulation also activates innate immunity by engaging PRRs like TLR7/8, triggering cytokine and Tfh cell activation essential for high-affinity neutralizing antibodies [20,92].

Self-assembling protein nanocages can be engineered by fusing antigens to the N- or C-terminus, a strategy compatible with mRNA, saRNA, taRNA, and circRNA vaccines (SAPN-RNA vaccines). This approach synergistically enhances vaccine efficacy by promoting higher and longer expression of antigenic proteins, stabilizing secreted proteins, improving their uptake by antigen-presenting cells, and increasing immunogenicity. SAPNs mimic viral structures without genetic material, enhancing B cell activation through highly repetitive antigen display. Their nanoscale size enables efficient APC uptake, prolonged antigen retention in lymph nodes, and Tfh cell activation, driving germinal center responses which enhances the generation of long-lived plasma cells and memory B cells [20]. As a result of these synergistic mechanisms of protein nanocages and the mRNA platform, SAPN-RNA vaccines can induce higher neutralizing antibody titers with cross-reactivity against heterogeneous strains, making them particularly beneficial for viruses undergoing rapid antigenic changes, such as influenza.

Incorporating SAPNs into RNA vaccine design not only enhances immunogenicity and dose efficiency, but also offers higher biocompatibility, due to their natural protein composition and favorable kinetic properties [15,35].

SAPNs such as ferritin have shown considerable promise in enhancing the immunogenicity of RNA vaccines, boosting both humoral and cellular immune responses. In the case of HIV-1, multivalent ferritin nanovaccines, such as those displaying the glycosylated RBD, have been shown to induce robust neutralizing antibody responses in mice, which effectively neutralized diverse HIV-1 strains [87].

The approach has demonstrated notable advantages, such as enhancing antigen expression and eliciting strong neutralizing responses [19]. Incorporating ferritin into an MERS-CoV RNA vaccine enabled broad cross-clade neutralization, including a pangolin-derived strain, and sustained immune responses for over five months post booster, underscoring their potential for durable, broad-spectrum protection [19].

Furthermore, SAPNs have been successfully applied in the development of mRNA vaccines for SARS-CoV-2. Ferritin-RBD fusion vaccines, in particular, elicited significantly higher neutralizing antibody levels compared to RBD alone, demonstrating a stronger humoral immune response in mouse models, with improved protection in hamsters [88].

LuS self-assembling nanocages enhance mRNA vaccine immunogenicity by presenting antigens in a virus-like 60-mer structure [38]. Phase 1 trials IAVI-G002 (NCT05001373) and IAVI-G003 (NCT05414786) demonstrated that LuS-mRNA vaccines were safe, and effectively induced broadly neutralizing antibody precursors with improved maturation and neutralization, validating this strategy in high-HIV-burden regions [23]. An mRNA vaccine encoding the Rotavirus P2-VP8 antigen displayed on LuS nanocages elicited the highest IgG and neutralizing antibody titers in mice and guinea pigs compared to conventional mRNA and subunit vaccines [89]. For SARS-CoV-2, the LuS-displayed multivalent RBD of the Delta variant induced potent neutralizing responses and conferred protection in mice [90]. The approach also enables bivalent vaccine designs, producing antibody responses comparable to monovalent formulations, highlighting its versatility [90].

Both non-replicating and self-amplifying mRNA vaccines rely on host-cell machinery to produce antigens and induce adaptive immunity. Non-replicating mRNA encodes only the target protein, whereas self-amplifying mRNA includes additional replication machinery, reducing the required dose for effective immunity [3]. Incorporating SAPNs can further enhance this by multivalent antigen display, improving cellular uptake, and enhancing expression and antigen presentation by APCs.

Besides conventional mRNA vaccines, this approach has been applied to self-amplifying RNA (saRNA) vaccines. Incorporation of LuS in saRNA induced robust anti-HIV Env antibody production and significantly enhanced antigen-specific B cell activation in mice compared to protein immunization, eliciting germinal center B and T follicular helper-cell responses with a balanced IgG1/IgG2 profile [24]. 

For bacteria, saRNA with ferritin-incorporation constructs encoding the F1 and V antigens of Yersinia pestis provided significant protection in mice against subsequent challenges. Mice immunized with 1 μg or 5 μg of saRNA in LNP showed survival (5/7 mice) against both low and high bacterial doses. This saRNA vaccine induced specific IgG responses and neutralizing antibodies, with the potential for reduced boosting and rapid production, making it a promising candidate for plague vaccine development and outbreak response [91]. Therefore, the SAPN-RNA approach can be applied to both non-replicating vaccines and self-replicating RNA vaccines. However, despite the potential of the approach, there is no data on taRNA and circRNA vaccines, and further exploration of these could synergize their efficacy.

Overall, the SAPN-RNA vaccine approach, i.e., integrating self-assembling protein nanocages into mRNA vaccines, enables multivalent antigen display, prolongs protein expression, reduces off-target effects, lowers dosing requirements, and enhances antigen stability, immunogenicity, and biocompatibility. Careful design is essential to prevent excessive innate immune activation and potential autoimmunity.

### 3.3. SAPN-DNA Vaccines: Integration with DNA Plasmids for Multivalent Antigen Display

DNA immunization is a promising vaccination strategy due to its stability, ease of design, and efficient plasmid production. However, unlike mRNA vaccines that act directly in the cytoplasm, DNA vaccines require nuclear entry, raising biosafety concerns under GMO regulations (Figure 2C). Moreover, DNA vaccines often exhibit lower immunogenicity compared to traditional platforms and typically require IM administration to achieve optimal efficacy, which has limited their broader use [107,108]. While immune modulators, cytokines, and costimulatory molecules have been investigated to enhance immune responses, these efforts have yielded only modest improvements [109].

The key challenge in optimizing DNA vaccines lies in enhancing transfection efficiency, as effective delivery and expression are crucial for inducing robust immune responses [110]. Technologies such as gene guns and electroporation have demonstrated potential for improving transfection rates and enhancing immune responses. However, these methods are not yet fully optimized for large-scale, routine applications [111].

SAPNs enhance DNA vaccine efficacy by fusing antigens to nanocages in DNA constructs, leveraging their nanostructure and multivalent surface display to improve cellular uptake, boost protein expression, stabilization and immunogenicity, and with potential for targeted delivery functionalization. For example, the SARS-CoV-2 glycosylated receptor-binding domain (gRBD) fused to *H. pylori* ferritin 24-mer elicited stronger neutralizing antibodies than the mi3 60-mer, highlighting the potential to enhance antibody responses and reduce vaccine doses and production costs [21].

Although nanoencapsulation techniques, such as chitosan nanoparticles [69,112] and live vectors [113], have proven effective for DNA vaccine delivery, early attempts to use protein nanocages for encapsulation of DNA likely encountered challenges [114,115]. The negative charge and large size of DNA plasmids seems to have hindered their efficient encapsulation and delivery, with electrostatic repulsion between negatively charged ferritin and the cell membrane limiting effective transfection.

Overall, SAPNs represent a promising approach to enhancing DNA vaccine efficacy. Their unique structural properties, such as the ability to multivalent display of antigens and the fact that they may encapsulate DNA for targeted delivery, allow for superior immunogenicity. As research progresses and challenges related to large-scale production and regulatory approval are addressed, SAPN-DNA vaccines could become a key component of modern vaccination strategies, offering substantial improvements in infectious disease prevention and cancer immunotherapy.

### 3.4. SAPN-Adjuvant: Multivalent Display of Adjuvant and Co-Delivery with Antigen

Beyond structural antigen presentation, SAPNs provide a versatile platform for the co-delivery of antigens and adjuvants, significantly enhancing APC activation and promoting robust immune responses [25,26,27]. The multivalent surface display of adjuvant proteins potentiates immune stimulation, while encapsulation of small-molecule adjuvants within the internal cavity of the nanocage allows for sustained release, prolonging vaccine efficacy and minimizing off-target effects [10,25,26,27]. These combined strategies enable precise immune modulation tailored to diverse pathogens.

The SAPN-Adjuvant approach utilizes the SAPNs to enhance immune responses by presenting multiple copies of protein-based adjuvants, such as interleukin cytokines, TLR agonists (e.g., flagellin), or other immune modulators, through various design strategies, including genetic fusion, chemical conjugation, and SpyTag/SpyCatcher [17]. However, reversible disassembly and genetic engineering of the platform’s internal cavity also enable encapsulation and co-delivery of these or small-molecule adjuvants [10].

Approaches include (1) multivalent surface display, (2) internal encapsulation, (3) a dual strategy where one adjuvant is surface-displayed and another encapsulated, and (4) co-delivery of an antigenic protein with an adjuvant, either both on the surface or with the antigen on the surface and adjuvant encapsulated. These configurations enable fine-tuning immune responses for Th1- or Th2-biased immunity or a balanced immune profile, depending on the pathogen. Additionally, the encapsulated or surface-displayed adjuvant using SAPNs can be easily mixed with an SAPN-based antigenic protein, further enhancing immune responses through combined administration. Protein-based adjuvants (PBAs) are potent immunomodulators, particularly for viral vaccines [116]. Flagellin, a TLR5 agonist and example of a PBA, stimulates APCs, promoting cytokine secretion and enhanced antigen uptake [117].

Conjugation of flagellin with influenza hemagglutinin on the exterior of ferritin nanocages significantly enhances both humoral and antigen-specific T cell responses, including Th1 cytokine production, and confers protection against lethal viral challenge in mice [26]. Similarly, genetic fusion of A-type flagellin (FliC) to ferritin nanocages generated the ReFliC-FN vaccine, which elicited a robust Th1-biased immune response and improved protection against *Pseudomonas aeruginosa*, while maintaining biocompatibility and safety [27]. Furthermore, immunization with Infectious Bronchitis Virus (IBV)-Flagellin SAPNs in chickens induced strong antibody responses, increased peripheral blood mononuclear-cell proliferation, and reduced viral shedding, demonstrating the approach’s efficacy across species [25].

Controlled release of encapsulated adjuvants enhances vaccine safety by reducing toxicity and promoting localized immune stimulation. SAPNs such as ferritin nanocages are effective delivery vehicles, due to their reversible disassembly; however, their small internal cavity (~8 nm) limits cargo capacity [17]. In contrast, larger encapsulin nanocages (20–42 nm) offer improved co-delivery of proteins, including antigens and adjuvants, with selective cargo loading mediated by targeting domains, thereby optimizing immune responses [118,119].

Effective incorporation of adjuvants into nanocage platforms requires careful consideration of their biological targets and activation mechanisms. For example, since flagellin is recognized by TLR5 on the extracellular membrane, internal encapsulation may limit direct receptor engagement. Truncated flagellin variants, such as the D0/D1 domains, have also been proposed to preserve TLR5 activation, while allowing efficient fusion and surface display on nanocage platforms due to their smaller size [120].

Overall, SAPN-Adjuvants provide a versatile approach for vaccine development, enabling precise immune modulation through tailored adjuvant presentation and delivery. By co-delivery with antigens through multivalent surface display or internal encapsulation, they hold potential to enhance immunogenicity, safety, and efficacy of vaccines.

## 4. SAPN-RNAi: Self-Assembling Protein Nanocages for RNAi Delivery

### 4.1. RNA Interference (RNAi) and Mechanisms

RNAi is a cellular mechanism that regulates gene expression by binding to complementary RNA, targeting mRNA, transposon intermediates, RNA viruses, and other types for degradation or translational repression [121]. The main RNAi pathways are mediated by small interfering RNAs (siRNAs) and microRNAs (miRNAs), typically 19–25 nucleotides in length with negative charge and molecular weight of ~14 kDa [122].

siRNAs are typically exogenous, double-stranded RNA molecules that are processed into smaller fragments by the enzyme Dicer. In contrast, miRNAs are endogenous, single-stranded RNAs that are derived from primary transcripts. Both siRNAs and miRNAs play significant roles in post-transcriptional regulation, though they function differently. siRNAs are highly specific and typically target a single mRNA for degradation, making them suitable for precise silencing, especially in therapeutic applications and viral infections. miRNAs, on the other hand, generally regulate the translation of multiple mRNAs by binding with partial complementarity, leading to broader modulation of gene expression [121,122,123].

The RNAi mechanism is conserved across many organisms and involves two main steps: initiation and effector. In siRNA-mediated RNAi, exogenous double-stranded RNA (dsRNA), which can originate from repetitive sequences or long hairpin structures, is processed by Dicer into 21–24 nucleotide siRNA duplexes. These duplexes, possessing 3′-OH, 5′ phosphate, and 3′ dinucleotide overhangs, then associate with Argonaute proteins to form the precursor RNA-induced silencing complex (pre-RISC). One strand of the siRNA duplex, the passenger strand, is cleaved by Argonaute, while the remaining guide strand directs the mature RISC complex to complementary mRNAs for cleavage and degradation [28,124].

In miRNA-mediated RNAi, the process initiates with the transcription of primary miRNAs (pri-miRNAs). These pri-miRNAs are processed by the Microprocessor complex, consisting of the enzyme Drosha and its cofactor DGCR8, into precursor miRNAs (pre-miRNAs). The pre-miRNAs are then exported from the nucleus to the cytoplasm by Exportin-5 in a Ran-GTP-dependent manner. In the cytoplasm, the RNase III enzyme Dicer further processes the pre-miRNAs into miRNA duplexes. One strand of the duplex, known as the guide strand, is incorporated into the RISC. The guide strand directs RISC to target mRNAs with partial complementarity, leading to translational repression or mRNA degradation, thereby enabling broader modulating gene activity [125].

Another type of RNAi molecule, short hairpin RNA (shRNA), is a synthetic RNA structure that mimics endogenous RNA precursors and is commonly used in stable gene knockdown experiments [126]. They form a hairpin structure, target specific genes, and are processed into siRNA by the RNAi machinery when introduced into cells via vectors, with first-generation shRNAs having off-target effects, while second-generation shRNAs offer greater adaptability and stability but remain more complex [127]. Additionally, piwi-interacting RNAs (piRNAs), 21–35 nt in length, regulate gene expression through PIWI proteins and are involved in transposon silencing and maintaining genome stability in germline cells by mediating both transcriptional and post-transcriptional silencing, with therapeutic potential [128].

**Figure 3:**
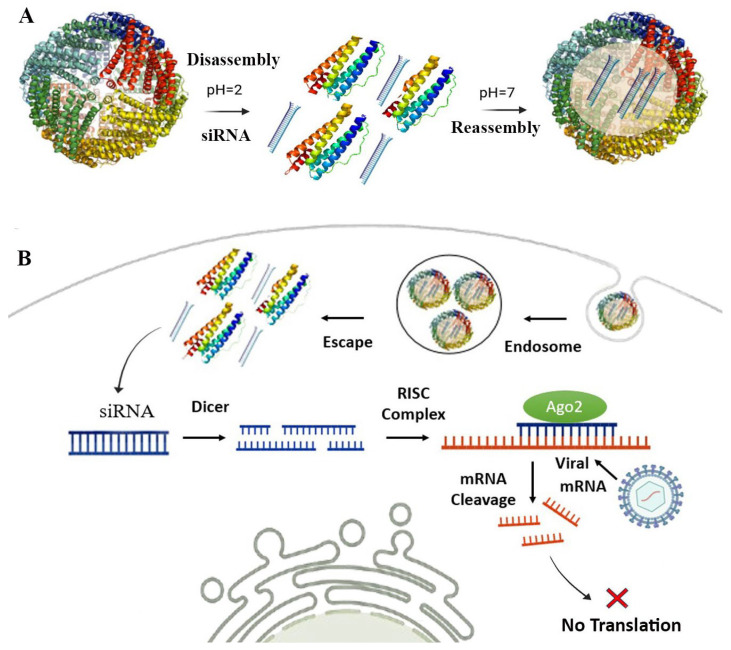
Schematic of SAPN-RNAi delivery for antiviral therapy. (**A**) Self-assembling protein nanocages (SAPNs) for RNAi encapsulation: siRNA is encapsulated within ferritin via reversible disassembly at pH 2 and reassembly at pH 7. (**B**) Antiviral mechanism of SAPN-RNAi: SAPN protects siRNA from degradation and facilitates cellular uptake via endocytosis. After endosomal escape, Dicer processes long dsRNA into siRNAs that guide Ago2-RISC to degrade viral mRNA and inhibit protein synthesis. Targeting-ligands on the SAPN surface ensure cell-specific delivery. Created with BioRender.com.

A key aspect of RNAi-based therapies is effective delivery. Various strategies have been developed to enhance delivery and minimize degradation [127]. LNPs can protect RNA from degradation and facilitate cellular uptake [129]. Viral vectors also offer efficient delivery but may raise concerns regarding immunogenicity and potential genome integration [130]. Synthetic polymers enhance stability and targeting with less toxicity, while physical methods like electroporation allow direct delivery but are invasive [127]. Despite these advancements, RNAi delivery remains a major challenge. The SAPN-RNAi delivery approach for antiviral therapy is shown in Figure 3.

### 4.2. Self-Assembling Protein Nanocages for RNAi Delivery Against Viral Infection

RNA interference provides a targeted therapeutic strategy for managing viral infections, offering greater adaptability than traditional antiviral approaches. siRNAs are particularly effective due to their high specificity, binding to and degrading viral RNA, thereby disrupting replication and limiting disease progression [6].

Once introduced into cells, siRNAs incorporate into RISC, where the guide strand directs cleavage of complementary viral mRNAs, preventing protein synthesis and viral propagation [5]. Additionally, synthetic siRNAs bypass the Dicer-mediated processing step and directly integrate into RISC to initiate gene silencing [131,132].

The efficacy of siRNAs in targeting chronic and lethal viruses, including HBV, HIV, influenza, and SARS-CoV, has been demonstrated, with promising results in inhibiting viral replication [124]. Currently, five siRNA-based drugs including patisiran, givosiran, inclisiran, lumasiran, and vurtisiran, have been approved, with others in late-stage clinical trials [133]. However, efficient delivery of RNAi molecules into target cells remains a major challenge, as effective delivery systems are needed to ensure sufficient RNA concentrations while minimizing off-target effects and immune responses [127].

Nanoparticles have proven to be effective siRNA delivery systems with minimal toxicity and off-target effects [134]. Among them, protein nanocages such as ferritin, offer a highly efficient and versatile platform for RNAi therapy. Their internal cavity enables siRNA encapsulation, while surface engineering allows targeted delivery. With excellent biosafety, rapid dispersion, and controlled release, SAPNs enhance therapeutic stability and efficacy [66,70]. Their well-defined nanostructures, structural stability, and functionalization capacity protect RNA from degradation and facilitate cellular uptake [13,29,30,31]. SAPNs are well-suited for delivering small RNA molecules such as siRNA and miRNA, rather than larger mRNA or DNA vaccines, as their size (5–8 nm) aligns with the protein nanocage cavity size and loading capacity [28,115].

The ability of SAPNs to undergo controlled disassembly and reassembly enhances their utility for RNAi delivery. For example, ferritin, a naturally occurring SAPN, can disassemble at acidic pH 2, allowing RNA loading, and reassemble at physiological pH (~7.4), encapsulating the RNA payload and protecting it from degradation [66]. This method enables flexible RNAi delivery while maintaining structural integrity. Alternative techniques, such as urea gradients and hydrostatic pressure, further facilitate RNA loading [9], providing versatile approaches for developing SAPN- RNAi delivery systems.

Encapsulation of siRNA within human apoferritin has demonstrated superior protection against enzymatic degradation and efficient cellular uptake. This system achieves high transfection efficiency in diverse cell types, including peripheral blood mononuclear cells (PBMCs), without inducing immune activation [29].

Such characteristics make SAPN-based RNAi delivery systems promising for antiviral therapies aimed at suppressing viral gene expression and modulating immune responses. Insights from ferritin-based antitumor delivery systems can inform their application in antiviral contexts. For instance, ferritin modified with the cell-penetrating peptide L17E demonstrated enhanced cellular uptake and stability relative to wild-type ferritin. When loaded with BCL-2 siRNA, this platform (Fn-L17E) effectively silenced the anti-apoptotic gene BCL-2 in A549 and HeLa cells [13]. Similarly, ferritin fused with the KALA peptide effectively targeted dendritic cells and enhanced antigen presentation. Co-delivery of SOCS1 siRNA further promoted DC–T cell interactions, resulting in durable antitumor immunity [67].

Additionally, siPD-L1/HFn ferritin nanocarriers have exhibited significant PD-L1 silencing in HL-60 and K-562 cancer cell lines, further supporting their promise in RNAi-based therapeutic strategies [30]. In targeted RNAi therapy for glioblastoma, the positively charged ferritin variant, tHFn(+), enhances lysosomal escape, facilitating the release of siRNA into the cytoplasm. Both in vitro and in vivo studies confirm that tHFn(+) efficiently crosses the blood–brain barrier (BBB) and specifically targets glioblastoma, with siTERT and siEGFR showing therapeutic efficacy in mouse models [31].

Encapsulins, with their larger size and superior cargo loading capacity, outperform other protein nanocages in delivering larger RNA molecules or multiple siRNA sequences for advanced antiviral therapies [118]. Unlike traditional systems requiring in vitro cargo loading, encapsulins enable in vivo co-packaging of RNA and proteins via selective loading mechanisms and targeting domains, preventing conditions that could destabilize RNA [11]. However, integrating a non-protein cargo like siRNA or mRNA requires additional engineering, such as RNA-binding domains or charge-based modifications like a cationic RKRK domain, to overcome electrostatic repulsion with the cell membrane and ensure effective transfection [11,115,135].

A key advantage of co-delivering protein and RNA within a single nanocage is the ability to target multiple intracellular pathways, thereby enhancing immune responses through antigen presentation and silencing of immune-suppressive pathways. Nanocages also protect RNA from degradation, with pH-sensitive disassembly facilitating efficient release. In vivo assembly further simplifies production and minimizes non-specific loading [11,42].

However, challenges such as optimizing loading capacity, preventing RNA degradation during pH modulation, and preserving the native structure of the protein nanocages remain critical. To address these issues, various ferritin mutants have been engineered to enhance pH-responsive disassembly/reassembly for drug delivery. Approaches include C-terminal modifications (e.g., GALA fusion, DE helix deletion), loop truncations (AB, DE loops), and histidine substitutions, to enable structural reversibility and cargo encapsulation [136]. Future advancements may improve RNA selectivity via sequence-specific RNA-binding domains. Notably, functionalizing SAPNs with viral-targeting ligands and refining assembly mechanisms could enable these systems for effective antiviral siRNA delivery.

Overall, the SAPN-RNAi approach shows promise for treating viral infections by targeting conserved viral genes or modulating host factors critical for viral replication. Advancements in delivery strategies, including the evaluation of various protein nanocages and loading techniques, could lead to next-generation antiviral therapies with improved specificity, stability, and efficacy.

## 5. Challenges and Future Direction

Self-assembling protein nanocages (SAPNs) occur naturally in various organisms, providing a versatile platform for vaccine development and therapeutic delivery, including RNAi. Their modular architecture enables precise immune modulation through multivalent antigen display, while the inner cavity supports cargo loading for enhanced stability, controlled release, with targeted delivery. The unique nanostructure improves cellular uptake and antigen presentation, and facilitates the co-delivery of antigens and adjuvants, optimizing immune responses. Incorporating SAPNs into nucleic acid vaccines platforms (mRNA and DNA) offers a novel strategy to synergize their immune responses at lower doses while enhancing stability, versatility and cross-protection against evolving pathogens. Encapsulation and protection of therapeutic RNAi with targeted delivery is another potential of this novel strategy. Approaches for vaccine design and RNAi delivery, along with offered corresponding terms, are summarized in Table 2.

Despite their potential, several challenges must be addressed to fully harness the benefits of SAPNs in vaccine development and RNAi therapeutics. Antigen subunits can be genetically fused to the C- or N-terminus of proteins; however, increasing antigen size and structural complexity may compromise nanoparticle assembly and integrity. To optimize antigen presentation, regardless of size, SAPNs designed for subunit vaccines may require modular engineering approaches, such as SpyTag-SpyCatcher systems. Larger protein nanocages or multi-epitopic/short transduced antigens, combined with suitable linkers, may be preferable to full-length antigens for nucleic acid-based vaccines (RNA and DNA vaccines). Excessive innate immune activation or autoimmunity must be considered, as they could impact antigen translation and RNAi delivery. Thus, biocompatibility studies and dose optimization using non-homologous protein nanocages, especially those derived from bacterial sources, are recommended. Importantly, protein nanocages should be screened for autoimmune epitopes and engineered to minimize risks.

Alongside multivalent antigen display, future research should focus on the encapsulation of mRNA vaccines using protein nanocages. Challenges related to charge properties, release kinetics, and structural integrity must be addressed. The negative charge of SAPNs presents a limitation, hindering RNA loading and delivery. This can be mitigated by engineering positively charged SAPN variants or incorporating nanoparticles like chitosan to enhance electrostatic interactions. Optimizing RNA encapsulation while maintaining controlled release is also critical. Advancements, such as pH- or enzyme-responsive disassembly mechanisms, could enhance RNA release at target sites. Furthermore, incorporating cell-specific ligands or fusion proteins could improve targeting and therapeutic efficacy. Hybrid delivery strategies, combining SAPNs with lipid or polymer nanocarriers, may also be applied to expand clinical applicability. Beyond RNAi delivery, SAPNs also hold promise for emerging nucleic acid therapeutics like antisense oligonucleotides (ASOs), which offer adaptable gene-silencing mechanisms

Selecting the appropriate SAPN scaffold for each application is crucial, as factors such as stability, flexibility, expression system compatibility, loading capacity, and subunit composition impact efficacy. Well-characterized nanocages, like ferritin, have proven useful for both antigen display and RNA encapsulation, while other protein nanocages, such as encapsulins, may offer advantages in size, subunit composition, and dual delivery potential. Nonetheless, further exploration of protein nanocages from diverse sources, along with extensive characterization, is necessary to validate this approach across a variety of pathogens and diseases in both humans and animals.

Comprehensive preclinical and clinical studies are thus essential to validate the safety and efficacy of SAPN platforms. Addressing regulatory challenges will be crucial to ensuring these innovative technologies meet stringent clinical approval standards. By optimizing SAPN design and production and overcoming these challenges, these platforms hold significant promise for advancing vaccines and RNAi therapeutics for viral infections and other diseases.

## Figures and Tables

**Table 1:** Characteristics of main Self-Assembling Protein Nanocages (SAPNs) used in Vaccine Development and RNAi Delivery.

SAPNs	Biological Function	Origin	Cavity Size (nm)	Outer Size (nm)	Subunits	Subunit MW	Stability	Ref.
Ferritin	Iron storage and transport	*Helicobacter pylori*	8	12	24	20 kDa	pH: 3–12; 80–100 °C	[17]
Lumazine Synthase (LuS)	Riboflavin biosynthesis	*Aquifex aeolicus*	9	15.4	60	17.8 kDa	120 °C	[38]
		*Bacillus subtilis*	16	30	180	16.3 kDa	Up to pH: 10 and 80 °C	[37]
Encapsulin	Oxidative stress response through encapsulation of other related proteins	*Thermotoga maritima*	20	24	60	31 kDa	pH: 12, <3 M GuHCl; 90 °C	[8,58]
		*Pyrococcus furiosus*	22	32	180	39 kDa	<4 M GuHCl; 80 °C	[8,58]
		*Quasibacillus thermotolerance*	36	42	240	32.2 kDa	<1 M GuHCl; 40 °C	[8,58]
E2p	Enzyme complex for metabolic pathways	*Geobacillus stearothermophilus*	12 pores of 5 nm	27	60	41 kDa	80 °C	[48]
sHSP	Response to cellular stress	*Methanococcus jannaschii*	6	12	24	16.5 kDa	70 °C and pH 5–11	[56]

**Table 2:** Innovative Strategies Employing Self-Assembling Protein Nanocages (SAPNs) in Vaccine Design and RNAi Therapy.

Approach	Developing Method	Key Advantages Compared to Conventional Method
SAPN-Subunit Vaccines	Fuse the antigen to the N- or C-termini of SAPN, express and purify the fusion protein to devlop the subunit vaccine.	Multivalent antigen display, enhanced solubility, stability and biocompatibility, cellular uptake, heightened immunogenicity, targeted delivery, and customized vaccine design.
SAPN-RNA Vaccines	Fuse the antigen to the N- or C-termini of SAPN, produce the antigen via RNA vaccine platforms (mRNA, saRNA, taRNA).	Promotes multivalent antigen display, enhances stability and immunogenicity of secreted proteins with prolonged expression, reduces off-target effects, and minimizes required doses.
SAPN-DNA Vaccines	Fuse the antigen to the N- or C-termini of SAPN, clone the fusion construct into a eukaryotic DNA plasmid.	Multivalent antigen display that stabilizes the antigenic protein and enhances expression, cellular uptake, antigen presentation, and immune responses.
SAPN-Adjuvant	Fuse a protein adjuvant to the N- or C-termini of SAPN, co-deliver with the antigen or encapsulate within SAPN.	Enables co-delivery of adjuvant with antigen to same cell (e.g., APC), targeted and enhanced biocompatibility, stability and immune responses with multivalent display of protein adjuvants, protection and controlled release of encapsulated small-molecule adjuvants.
SAPN-RNAi Delivery	Encapsulate siRNA or miRNA within SAPN using the disassembly/reassembly of SAPN.	Enables targeted gene silencing, controlled release, enhanced delivery efficiency, reduced immunogenicity, and scalable production.

**Table 3:** Self-Assembling Protein Nanocages (SAPNs) for Nucleic Acid Vaccine (mRNA and DNA) Development and RNAi Delivery.

Platform	SAPN Type	Mode of Incorporation	Diseases/Pathogen	Species/Cells	Key Finding	Ref.
mRNA vaccine	Ferritin	Genetic Surface Fusion	MERS-CoV	Mice; NHPs and Alpacas	This vaccine is temperature- and pH-stable, eliciting robust antibody titers in BALB/c mice and NHPs (non-human primates) with cross-clade neutralization (A, B, C). Complete protection in alpacas.	[19]
mRNA vaccine	Ferritin	Genetic Surface Fusion	HIV-1 Env	Mice	Induced neutralizing antibody responses in mice capable of neutralizing heterologous HIV-1 isolates.	[87]
mRNA vaccine	Ferritin	Genetic Surface Fusion	SARS-CoV-2 (RBD)	Mice and Hamsters	Elevated specific IgG levels and significantly higher neutralizing antibody titers correlated with improved protection in hamsters.	[88]
mRNA vaccine	LuS	Genetic Surface Fusion	Rotavirus (P2-VP8*)	Mice and Guinea Pigs	Induced the highest specific IgG titers compared to conventional mRNA and subunit vaccine.	[89]
mRNA vaccine	LuS	Genetic Surface Fusion	SARS-CoV-2 (RBD)	Mice	Strong neutralizing antibody responses and protection against the Delta variant.	[90]
mRNA vaccine	LuS	Genetic Surface Fusion	HIV (gp120)	Human	The vaccines were safe and induce early maturation of HIV broadly neutralizing antibody (bnAb) precursors in humans. Phase 1 clinical trial (NCT05001373 and NCT05414786).	[23]
saRNA vaccine	LuS	Genetic Surface Fusion	HIV (gp120)	Mice	Elicited enhanced Tfh cell activation, increased B cell responses, and stronger antibody responses in mice compared to protein vaccines	[24]
saRNA vaccine	Ferritin	Genetic Surface Fusion	Yersinia pestis	Mice	Induced specific IgG responses and neutralizing antibodies, conferring protection in mice against *Yersinia pestis* challenge.	[91]
DNA vaccine	Ferritin	Genetic Surface Fusion	SARS-CoV-2 (RBD)	Rat	Generates more potent neutralizing responses compared to the subunit vaccine.	[21]
siRNA	Ferritin	Reversible dissassembly	Cancer	hMSC and PBMCs cells	Efficient encapsulation enabling gene silencing in tumor cells and PBMCs at low concentrations without inducing immune activation.	[29]
siRNA	Ferritin	Reversible dissassembly	Acute myeloid leukemia	HL-60 and K-562	The siPD-L1/HFn nanocarrier effectively silenced PD-L1 expression in HL-60 and K-562 cells.	[30]
siRNA	Ferritin	Reversible dissassembly	Cancer	A549 and HeLa cells	L17E-modified ferritin boosts stability, uptake, and BCL-2 silencing in A549 and HeLa cells.	[13]
siRNA	Ferritin (tHFn(+))	Reversible dissassembly	Glioblastoma	Mice	Efficiently crosses the blood–brain barrier, targets glioblastoma, and demonstrates therapeutic efficacy with siTERT and siEGFR in mice.	[31]

Note: In vaccines, SAPNs are genetically fused to antigens for multivalent display; in RNAi therapies, they encapsulate siRNA via reversible disassembly.
